# One new species of the genus *Ischnothyreus* Simon, 1893 and re-description of *I. yueluensis* Yin & Wang, 1984 from China (Araneae, Oonopidae)

**DOI:** 10.3897/BDJ.9.e66843

**Published:** 2021-05-11

**Authors:** Ying Huang, Yanfeng Tong, Dongju Bian, Shuqiang Li

**Affiliations:** 1 College of Life Sciences, Shenyang Normal University, Shenyang, China College of Life Sciences, Shenyang Normal University Shenyang China; 2 Key Laboratory of Forest Ecology and Management, Institute of Applied Ecology, Chinese Academy of Sciences, Shenyang, China Key Laboratory of Forest Ecology and Management, Institute of Applied Ecology, Chinese Academy of Sciences Shenyang China; 3 Institute of Zoology, Chinese Academy of Sciences, Beijing, China Institute of Zoology, Chinese Academy of Sciences Beijing China

**Keywords:** biodiversity, goblin spiders, new species, taxonomy

## Abstract

**Background:**

The genus *Ischnothyreus* Simon, 1893 is one of the most speciose genera of Oonopidae, with 114 extant species mainly distributed in the Old World. Currently, 16 species have been recorded in China.

**New information:**

Two species of the genus *Ischnothyreus* Simon, 1893 from China are recognised, including one new species, *I. yunlong* Tong & Li, sp. n. (male, female) from Yunnan. *Ischnothyreus yueluensis* Yin & Wang, 1984 is re-studied. Descriptions, diagnoses and photos of the two species are provided.

## Introduction

*Ischnothyreus* Simon, 1893 is one of the most speciose genera of Oonopidae, with 114 extant species mainly distributed in the Old World (WSC 2021). This genus is currently placed in the subfamily Oonopinae as they have the characteristic tarsal organ pattern found in this group (Platnick et al. 2012). Members of this genus can easily be recognised by the strong spines present on the femora, tibiae and metatarsi of the first and second legs in both sexes, the distinctive, small, darkened male palps and the distinct, darkly sclerotised, convoluted duct and uniquely-shaped atrium in females (Kranz-Baltensperger 2011, Edward and Harvey 2014).

Up to now, 16 species of the genus *Ischnothyreus* have been recorded in China, of these, eight species, i.e. *I. auritus* Tong & Li, 2012, *I. campanaceus* Tong & Li, 2008, *I. falcatus* Tong & Li, 2008, *I. hanae* Tong & Li, 2008, *I. qianlongae* Tong & Li, 2008, *I. spineus* Tong & Li, 2012, *I. xui* Tong & Li, 2012 and *I. yuanyeae* Tong & Li, 2012, are only known from Hainan; *I. flagellichelis* Xu, 1989 is only known from Anhui; *I. kentingensis* Tong & Li, 2014 is only known from Taiwan; *I. linzhiensis* Hu, 2001 is only known from Tibet; *I. tergemintus* Liu, Xu & Henrard, 2019 is only known from Jiangxi; *I. zhoujiayan* Tong & Li, 2018 is only known from Chongqing; *I. narutomii* (Nakatsudi, 1942) is known from Hainan and Taiwan and *I. peltifer* (Simon, 1892) and *I. yueluensis* Yin & Wang, 1984 are known from south China (Tong and Li 2008, Tong and Li 2012, Tong and Li 2014, Tong et al. 2018, Liu et al. 2019). In this paper one new species of *Ischnothyreus*, collected from Yunnan, is reported and a detailed re-description of *Ischnothyreus yueluensis* Yin & Wang, 1984 is provided.

## Materials and methods

Specimens in this study were mainly collected by sieving from forest leaf-litter. The specimens were first examined in 95% ethanol using a Leica M205C stereomicroscope. Details were then studied with an Olympus BX51 compound microscope. Photos were taken with a Canon EOS 750D zoom digital camera (18 megapixels), mounted on an Olympus BX51 compound microscope and Helicon Focus image stacking software (7.6.1 Lite) was used to compile the images. Vulvae were cleared in lactic acid, then immersed in Kaiser's glycerol gelatine for photographs. Scanning electron microscope images (SEM) were taken under high vacuum with a Hitachi TM3030 after critical point drying and gold-palladium coating. All measurements were taken using an Olympus BX51 compound microscope and are given in millimetres in the text.

The specimens are preserved in Shenyang Normal University (SYNU) in Shenyang, China and Hunan Normal University (HNNU), Changsha, China.

The following abbreviations are used in the text and figures: a = apodemes; ALE = anterior lateral eyes; bse = bell-shaped sclerotised extension; dlm = dorsal leaf-shaped membrane; dp = dorsal protuberance; fp = flag-shaped process; pl = prolateral lobe; PLE = posterior lateral eyes; plm = prolateral leaf-shaped membrane; PME = posterior median eyes; rl = retrolateral lobe; sp = stick-shaped process; ssp = small sclerotised process; ste = sharp, tooth-like extension; stp = strong, tooth-like projection; vp = ventral projection; vpr = ventral protuberances; wt = winding tube.

## Taxon treatments

### Ischnothyreus yunlong

Tong & Li sp. n.

CB46DF85-1CF0-582A-BEC6-E99E71C8ACAB

6643B644-4C98-46C5-BD10-6F70AB37E847

#### Materials

- **Type status:** Holotype. **Occurrence:** individualID: SYNU-478; individualCount: 1; sex: male; lifeStage: adult; preparations: whole animal; disposition: in collection; **Taxon:** scientificName: *Ischnothyreus yunlong*; order: Araneae; family: Oonopidae; genus: Ischnothyreus; **Location:** country: China; countryCode: CHN; stateProvince: Yunnan Province; county: Jianshui; locality: Yunlong Mountain Scenic Area; verbatimElevation: 1939 m a.s.l.; verbatimLatitude: 23º47.049'N; verbatimLongitude: 102º48.973'E; **Identification:** identifiedBy: Yanfeng Tong; **Event:** eventDate: 28 May 2015
- **Type status:** Paratype. **Occurrence:** individualID: SYNU-475–477; individualCount: 3; sex: female; lifeStage: adult; preparations: whole animal; **Taxon:** scientificName: *Ischnothyreus yunlong*; order: Araneae; family: Oonopidae; genus: Ischnothyreus; **Location:** country: China; countryCode: CHN; stateProvince: Yunnan Province; county: Jianshui; locality: Yunlong Mountain Scenic Area; verbatimElevation: 1939 m a.s.l.; verbatimLatitude: 23º47.049'N; verbatimLongitude: 102º48.973'E; **Identification:** identifiedBy: Yanfeng Tong; **Event:** eventDate: 28 May 2015

#### Description

**Figure 1.**
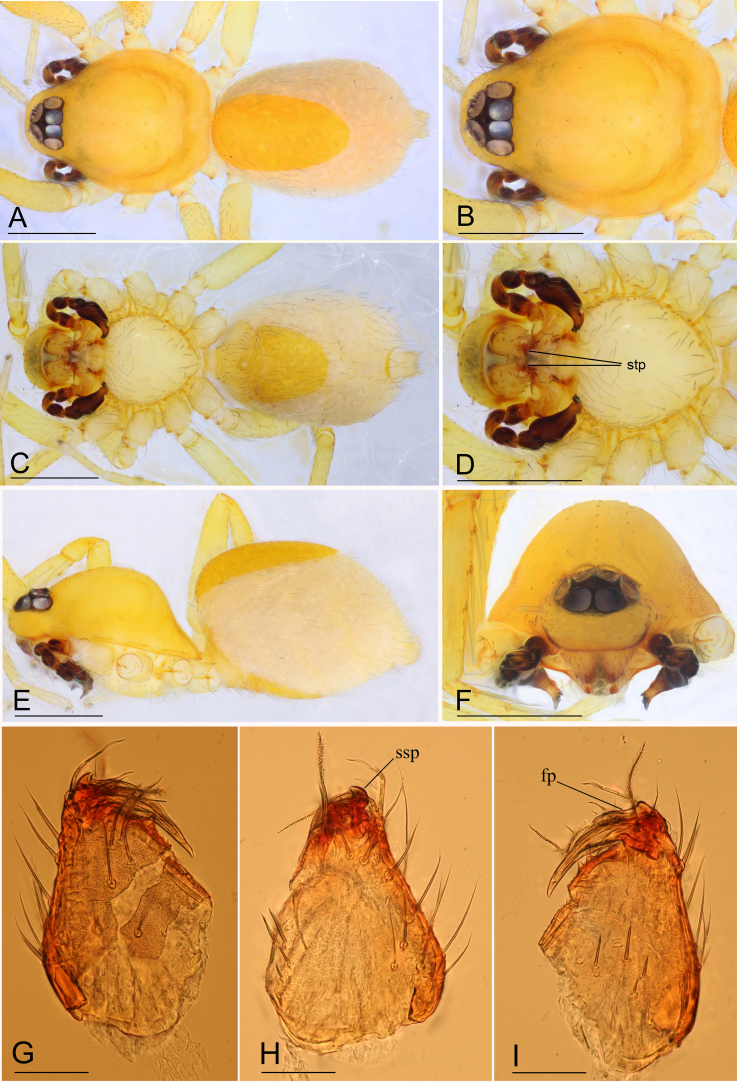
*Ischnothyreus yunlong* sp. n., holotype male. **A.** habitus, dorsal view; **B.** prosoma, dorsal view; **C.** habitus, ventral view; **D.** prosoma, ventral view; **E.** habitus, lateral view; **F.** prosoma, anterior view; **G.** left chelicerae, anterior view; **H.** left chelicerae, lateral view; **I.** left chelicerae, posterior view. Abbreviations: fp = flag-shaped process, ssp = small sclerotised process, stp = strong, tooth-like projection. Scale bars: 0.4 (A–F) and 0.05 (G–I).

**Figure 2.**
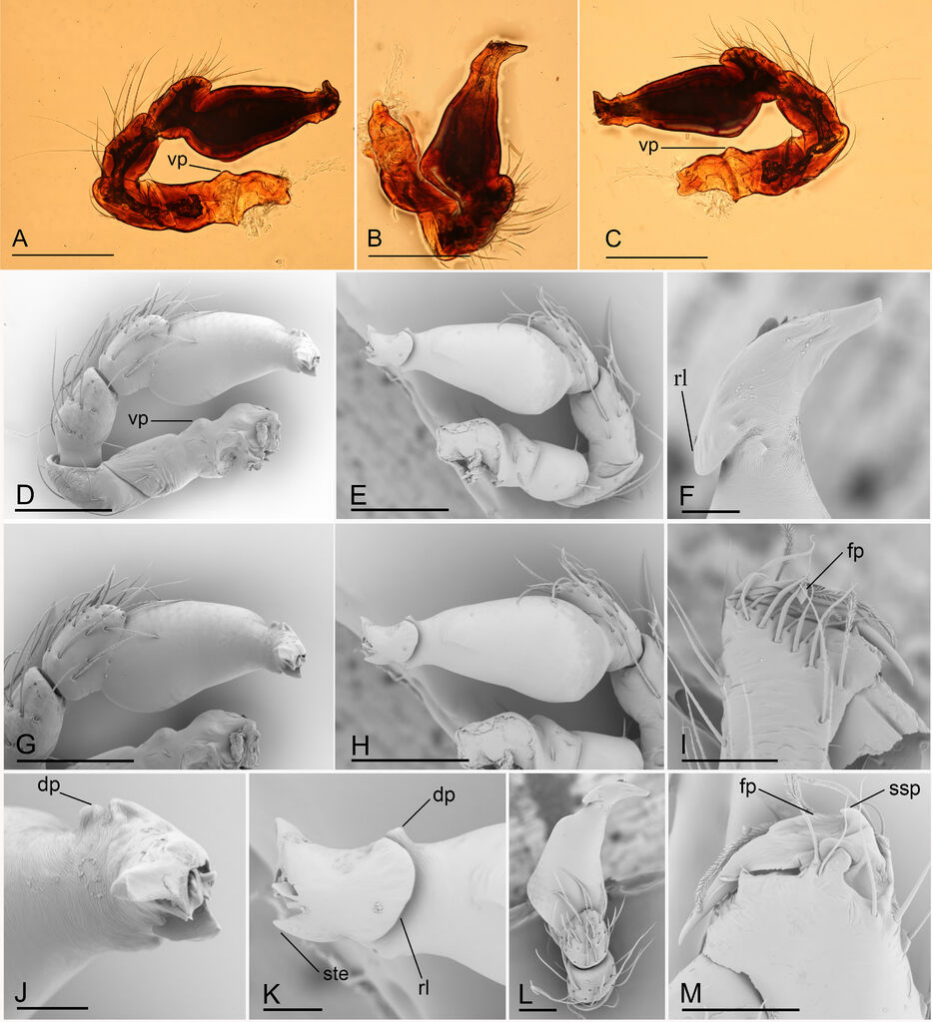
*Ischnothyreus yunlong* sp. n., holotype male, light (A–C) and SEM (D–M) microphotographs **A.** left palp, prolateral view; **B.** left palp, dorsal view; **C.** left palp, retrolateral view; **D.** left palp, prolateral view; **E.** left palp, retrolateral view; **F.** distal part of palpal bulb, dorsal view; **G.** palpal bulb, prolateral view; **H.** palpal bulb, retrolateral view; **I.** left chelicerae, anterior view; **J.** distal part of palpal bulb, prolateral view; **K.** distal part of palpal bulb, retrolateral view; **L.** palpal bulb, dorsal view; **M.** left chelicerae, posterior view. Abbreviations: dp = dorsal protuberance, fp = flag-shaped process, rl = retrolateral lobe, ssp = small sclerotised process, ste = sharp, tooth-like extension, vp = ventral projection. Scale bars: 0.1 (A–E, G, H), 0.05 (I, L, M) and 0.02 (F, J, K).

**Male (holotype)** (in mm). Body: habitus as in Fig. 1A, C and E; body length 1.75. Carapace (Fig. 1B and E): 0.85 long, 0.67 wide; yellow, with faint egg-shaped patches behind eyes, ovoid in dorsal view, profile dome-shaped, pars cephalica smooth, pars thoracica finely reticulate. Clypeus (Fig. 1F): rounded, slightly protruding, height about 1.45 times ALE diameter. Eyes (Fig. 1B and F): six, well developed, ALE largest, PME smallest, posterior eye row straight from above, procurved from front. Sternum (Fig. 1D): pale yellow, as long as wide. Mouthparts (Fig. 1D and G–I, Fig. 2I and M): chelicerae, endites and labium orange; chelicerae short, with large base, almost as wide as long, slightly recessed, fang with a flag-shaped process on proximal 1/3 and a small sclerotised process basally, fang groove with one large and a few small denticles; labium rounded, with sclerotised lateral margins; endites stout, anteromedian tip of endites with one strong, tooth-like projection. Abdomen: 0.91 long, 0.63 wide; dorsal scutum pale orange, oval, well sclerotised, covering 1/2 of abdomen width and approximately 3/5 of abdomen length, unfused to epigastric scutum; epigastric and postgastric scutum well sclerotised, pale orange, fused, postgastric scutum covering about 3/5 of abdomen length; spinneret scutum present, incomplete ring. Legs: pale orange, femur I with 2 prolateral spines, tibia I with 4 pairs, metatarsus I with 2 pairs of long ventral spines. Leg II spination similar to leg I, except femur with only 1 prolateral spine. Legs III and IV spineless. Sperm pore large, round, situated in front of anterior spiracles. Palp (Fig. 2A–H and J–L): trochanter with ventral projection; femur normal size; patella about as long as femur, not enlarged; tibia with three trichobothria; cymbium fused with bulb; bulb simple, without distinct ventral protuberance, distal end elongated, with a sharp tooth-like extension, with a small dorsal protuberance and a broad, rectangular-shaped retrolateral lobe.

**Figure 3.**
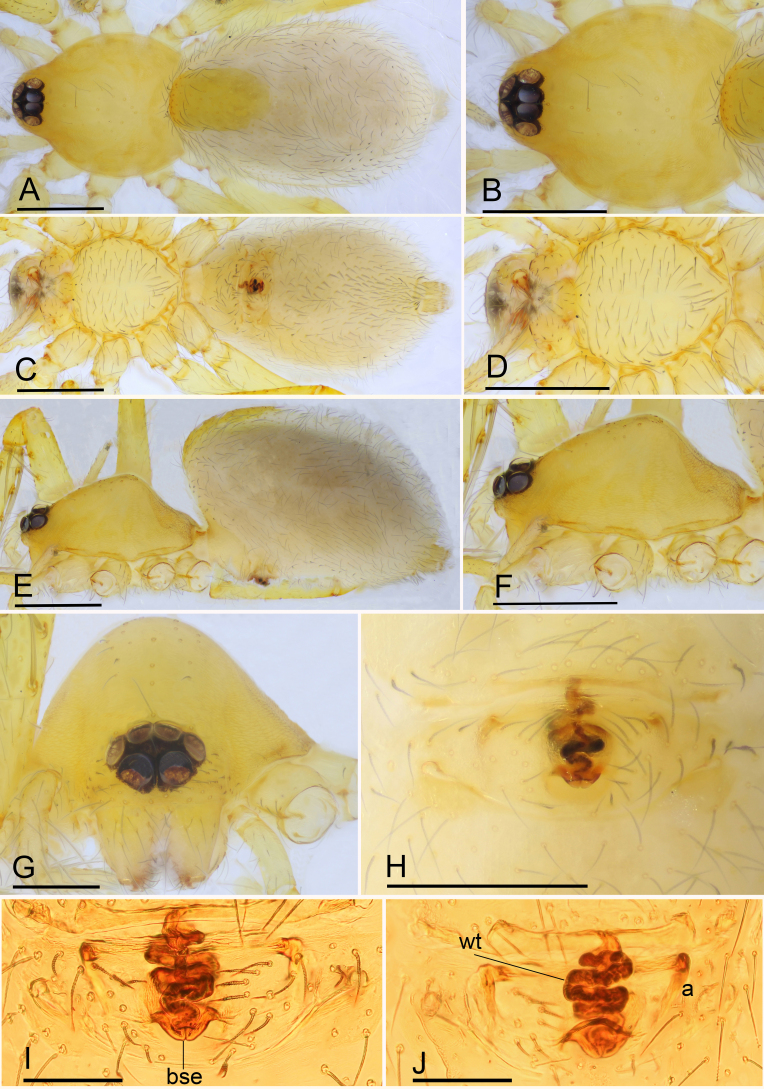
*Ischnothyreus yunlong* sp. n., paratype female **A.** habitus, dorsal view; **B.** prosoma, dorsal view; **C.** habitus, ventral view; **D.** prosoma, ventral view; **E.** habitus, lateral view; **F.** prosoma, lateral view; **G.** prosoma, anterior view; **H.** epigastric region, ventral view; **I.** endogyne, ventral view; **J.** endogyne, dorsal view. Abbreviations: a = apodemes, bse = bell-shaped sclerotised extension, wt = winding tube. Scale bars: 0.4 (A–F), 0.2 (G, H) and 0.1 (I, J).

**Female** (SYNU-475) (in mm): same as male, except as noted. Body: habitus as in Fig. 3A, C and E; body length 1.88. Carapace: 0.82 long, 0.68 wide, profile elevated. Clypeus lower, not protruding. Mouthparts: chelicerae unmodified; endite with serrula. Abdomen: 1.16 long, 0.77 wide; dorsal scutum covering 1/3 of abdomen length, about 1/4 of abdomen width. Epigastric area (Fig. 3H and I): surface without external features. Endogyne (Fig. 3J): from middle of slightly thickened margin of postgastric scutum runs a dark, tight complex winding tube, ending in a large bell-shaped sclerotised extension; posteriorly directed apodemes present.

#### Diagnosis

The new species is similar to *Ischnothyreus bauri* Richard, 2016 (female unknown) in the large retrolateral lobe of the palpal bulb, but can be distinguished by the absence of the ventral protuberance (Fig. 2G and H) of the palpal bulb (vs. two ventral protuberances (Richard et al. 2016: figs. 5A and B)) and the flag-shaped process (Fig. 2I and M) on proximal 1/3 of the male chelicerae fang and the small sclerotised process of fang base (vs. strongly thickened on proximal 2/3 and unmodified fang base (Richard et al. 2016: figs. 6A, B and C)). Females of the new species are similar to *Ischnothyreus campanaceus* Tong & Li, 2008, but can be distinguished by the small abdominal dorsal scutum (covering 1/3 of the abdomen length (Fig. 3A) vs. nearly 5/6 of the abdomen length (Tong 2013: fig. 44B)).

#### Etymology

The specific name is a noun in apposition from the type locality.

#### Distribution

Known only from the type locality (Fig. 7).

### Ischnothyreus yueluensis

Yin & Wang, 1984

C57377BD-4F7B-5362-BFB9-0D47127491CE

#### Materials

- **Type status:** Other material. **Occurrence:** individualID: SYNU-450–454; individualCount: 5; sex: 2 males, 3 females; lifeStage: adult; preparations: whole animal; disposition: in collection; **Taxon:** scientificName: *Ischnothyreus yueluensis*; namePublishedIn: Yin and Wang, 1984; order: Araneae; family: Oonopidae; genus: Ischnothyreus; **Location:** country: China; countryCode: CHN; stateProvince: Guangxi Zhuang Autonomous Region; county: Chongzuo City, Pingxiang City; locality: Nan Station, Sanzhi Cave; verbatimElevation: 257 m a.s.l.; verbatimLatitude: 22º04.540'N; verbatimLongitude: 106º44.264'E; **Identification:** identifiedBy: Yanfeng Tong; **Event:** eventDate: 7 May 2015
- **Type status:** Other material. **Occurrence:** individualID: SYNU-455; individualCount: 1; sex: female; lifeStage: adult; preparations: whole animal; disposition: in collection; **Taxon:** scientificName: *Ischnothyreus yueluensis*; namePublishedIn: Yin & Wang, 1984; order: Araneae; family: Oonopidae; genus: Ischnothyreus; **Location:** country: China; countryCode: CHN; stateProvince: Guangxi Zhuang Autonomous Region; county: Hechi City, Fengshan County; locality: Fengcheng Town, Songren Village, Xi’an Cave; verbatimElevation: 574 m a.s.l.; verbatimLatitude: 24º34.272'N; verbatimLongitude: 107º02.940'E; **Identification:** identifiedBy: Yanfeng Ton; **Event:** eventDate: 27 September 2015
- **Type status:** Other material. **Occurrence:** individualID: SYNU-456–458; individualCount: 3; sex: female; lifeStage: adult; preparations: whole animal; disposition: in collection; **Taxon:** scientificName: *Ischnothyreus yueluensis*; namePublishedIn: Yin & Wang, 1984; order: Araneae; family: Oonopidae; genus: Ischnothyreus; **Location:** country: China; countryCode: CHN; stateProvince: Guangxi Zhuang Autonomous Region; county: Nanning City; locality: Suxu Town, Mu Village, outside the Eighteen Luohan Cave; verbatimElevation: 196 m a.s.l.; verbatimLatitude: 22º32.600'N; verbatimLongitude: 108º03.390'E; **Identification:** identifiedBy: Yanfeng Tong; **Event:** eventDate: 9 May 2015
- **Type status:** Other material. **Occurrence:** individualID: SYNU-474; individualCount: 1; sex: male; lifeStage: adult; preparations: whole animal; disposition: in collection; **Taxon:** scientificName: *Ischnothyreus yueluensis*; namePublishedIn: Yin & Wang, 1984; order: Araneae; family: Oonopidae; genus: Ischnothyreus; **Location:** country: China; countryCode: CHN; stateProvince: Guangxi Zhuang Autonomous Region; county: Hechi City, Donglan County; locality: Simeng Town, an unnamed Cave; verbatimElevation: 464 m a.s.l.; verbatimLatitude: 24º30.071'N; verbatimLongitude: 107º16.181'E; **Identification:** identifiedBy: Yanfeng Tong; **Event:** eventID: 19 March 2015
- **Type status:** Other material. **Occurrence:** individualID: SYNU-459; individualCount: 1; sex: female; lifeStage: adult; preparations: whole animal; disposition: in collection; **Taxon:** scientificName: *Ischnothyreus yueluensis*; namePublishedIn: Yin & Wang, 1984; order: Araneae; family: Oonopidae; genus: Ischnothyreus; **Location:** country: China; countryCode: CHN; stateProvince: Guangxi Zhuang Autonomous Region; county: Guilin City; locality: Qixing Park, Qixingyan; verbatimElevation: 257 m a.s.l.; verbatimLatitude: 25º16.330'N; verbatimLongitude: 110º18.25'E; **Identification:** identifiedBy: Yanfeng Tong; **Event:** eventDate: 7 January 2013
- **Type status:** Other material. **Occurrence:** individualID: HNNU-YLS-17-0210; individualCount: 2; sex: 1 male, 1 female; lifeStage: adult; preparations: whole animal; disposition: in collection; **Taxon:** scientificName: *Ischnothyreus yueluensis*; namePublishedIn: Yin & Wang, 1984; order: Araneae; family: Oonopidae; genus: Ischnothyreus; **Location:** country: China; countryCode: CHN; stateProvince: Hunan Province; county: Changsha City; locality: Yuelu Mountain Scenic Area; verbatimElevation: 151 m a.s.l.; verbatimLatitude: 28º11.146'N; verbatimLongitude: 112º56.514'E; **Event:** eventDate: 17 February 2017

#### Description

**Figure 4.**
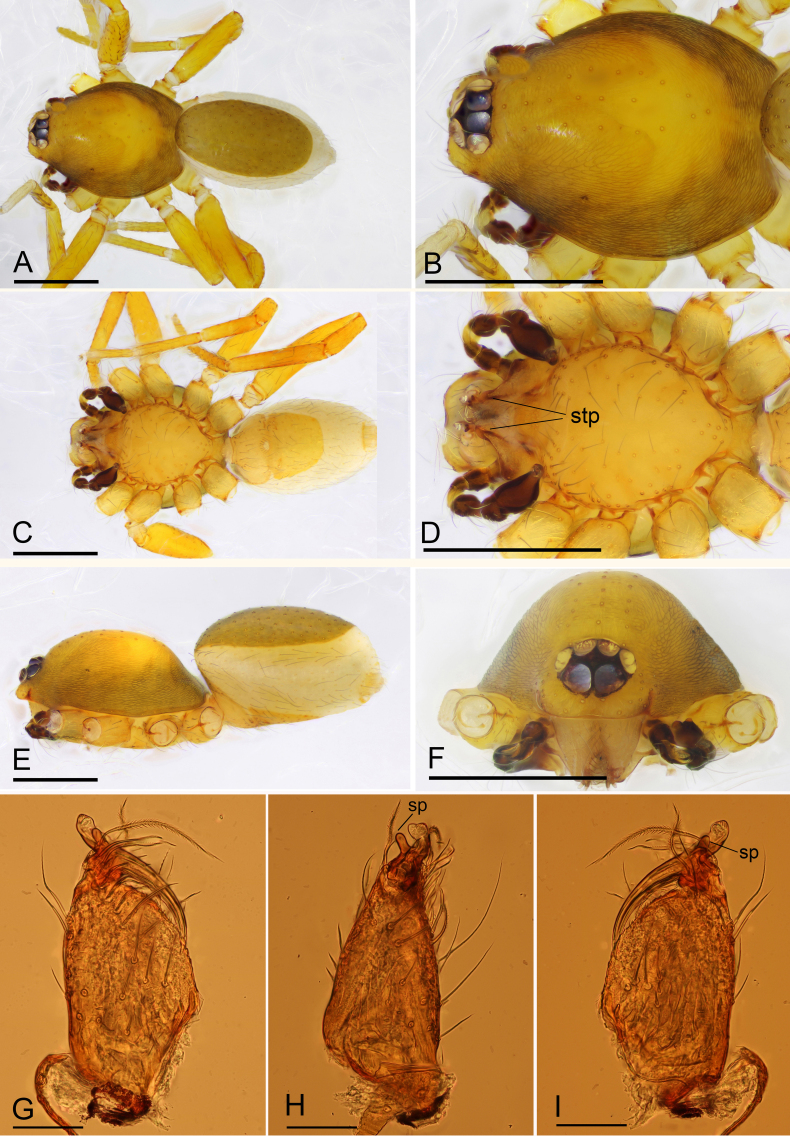
*Ischnothyreus yueluensis* Yin & Wang, 1984, male (SYNU-450) **A.** habitus, dorsal view; **B.** prosoma, dorsal view; **C.** habitus, ventral view; **D.** prosoma, ventral view; **E.** habitus, lateral view; **F.** prosoma, anterior view; **G.** left chelicerae, anterior view; **H.** left chelicerae, lateral view; **I.** left chelicerae, posterior view. Abbreviations: sp = stick-shaped process, stp = strong, tooth-like projection. Scale bars: 0.4 (A–F) and 0.05 (G–I).

**Figure 5.**
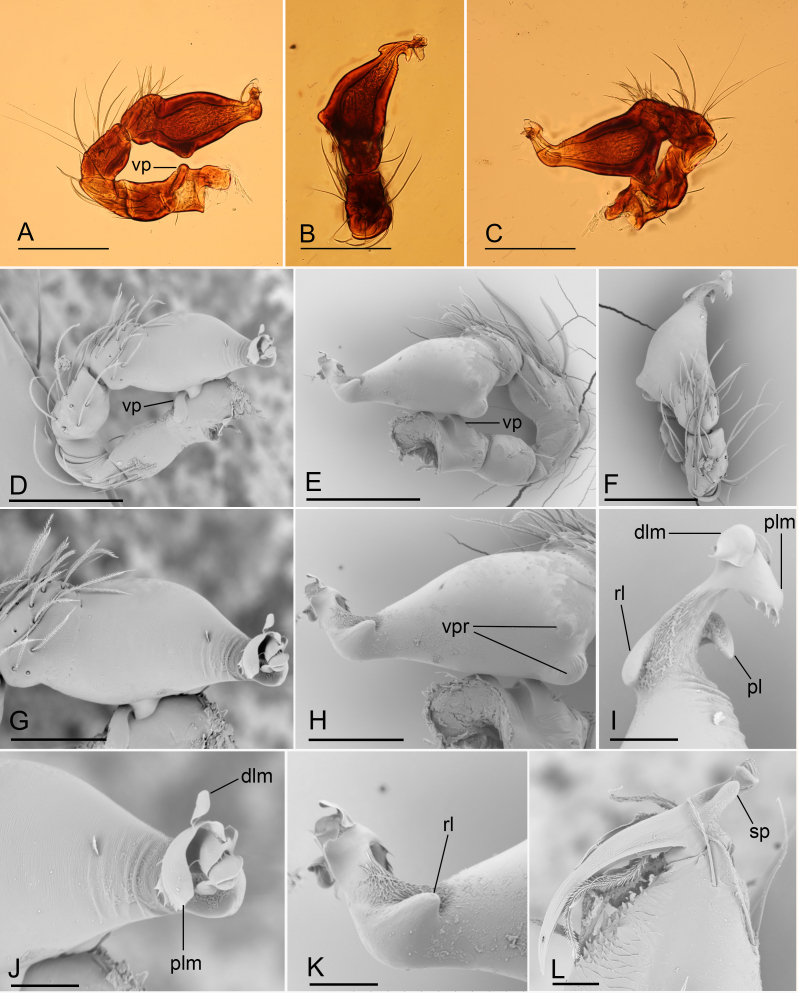
*Ischnothyreus yueluensis* Yin & Wang, 1984, male (SYNU-450), light (A–C) and SEM (D–L) microphotographs. **A.** left palp, prolateral view; **B.** left palp, dorsal view; **C.** left palp, retrolateral view; **D.** left palp, prolateral view; **E.** left palp, retrolateral view; **F.** left palp, dorsal view; **G.** palpal bulb, prolateral view; **H.** palpal bulb, retrolateral view; **I.** distal part of palpal bulb, dorsal view; **J.** distal part of palpal bulb, prolateral view; **K.** distal part of palpal bulb, retrolateral view; **L.** left chelicerae, posterior view. Abbreviations: dlm = dorsal leaf-shaped membrane, pl = prolateral lobe, plm = prolateral leaf-shaped membrane, rl = retrolateral lobe, sp = stick-shaped sclerotised process, vp = ventral projection, vpr = ventral protuberances. Scale bars: 0.1 (A–F), 0.05 (G, H) and 0.02 (I–L).

**Male** (SYNU-450) (in mm). Body: habitus as in Fig. 4A, C and E; body length 1.45. Carapace (Fig. 4B, E and F): 0.76 long, 0.56 wide; brown, with egg-shaped patches behind eyes, ovoid in dorsal view, profile elevated, surface of elevated portion of pars cephalica smooth, sides strongly reticulate, lateral margins straight, smooth. Clypeus (Fig. 4F): rounded, slightly protruding, height about 0.80 times of ALE diameter. Eyes (Fig. 4B and F): six, well developed, ALE largest, PLE smallest, posterior eye row straight from above, procurved from front. Sternum (Fig. 4D): pale orange, longer than wide. Mouthparts (Fig. 4D and G–I; Fig. 5L): chelicerae, endites and labium orange; chelicerae straight, base of fangs with small stick-shaped sclerotised process, fang groove with field of small denticles; anteromedian tip of endites with one strong, tooth-like projection. Abdomen: 0.75 long, 0.46 wide; dorsal scutum well sclerotised, pale orange, covering 4/5 of abdomen width and approximately 5/6 of abdomen length, unfused to epigastric scutum; epigastric and postgastric scutum well sclerotized, pale orange, fused, postgastric scutum covering about 2/3 of abdomen length. Legs: pale orange, femur I with 2 prolateral spines, tibia I with 4 pairs, metatarsus I with 2 pairs of long ventral spines. Leg II spination similar to leg I, except femur with only 1 prolateral spine. Legs III and IV spineless. Sperm pore large, oval, situated at level of anterior spiracles. Palp (Fig. 5A–K): trochanter with ventral projection; femur normal size; patella about as long as femur, not enlarged; tibia with three trichobothria; cymbium fused with bulb; bulb with 2 ventral protuberances, one large and another very small, distal end elongated, with a prolateral lobe and several leaf-shaped membranes, retrolateral lobe small.

**Figure 6.**
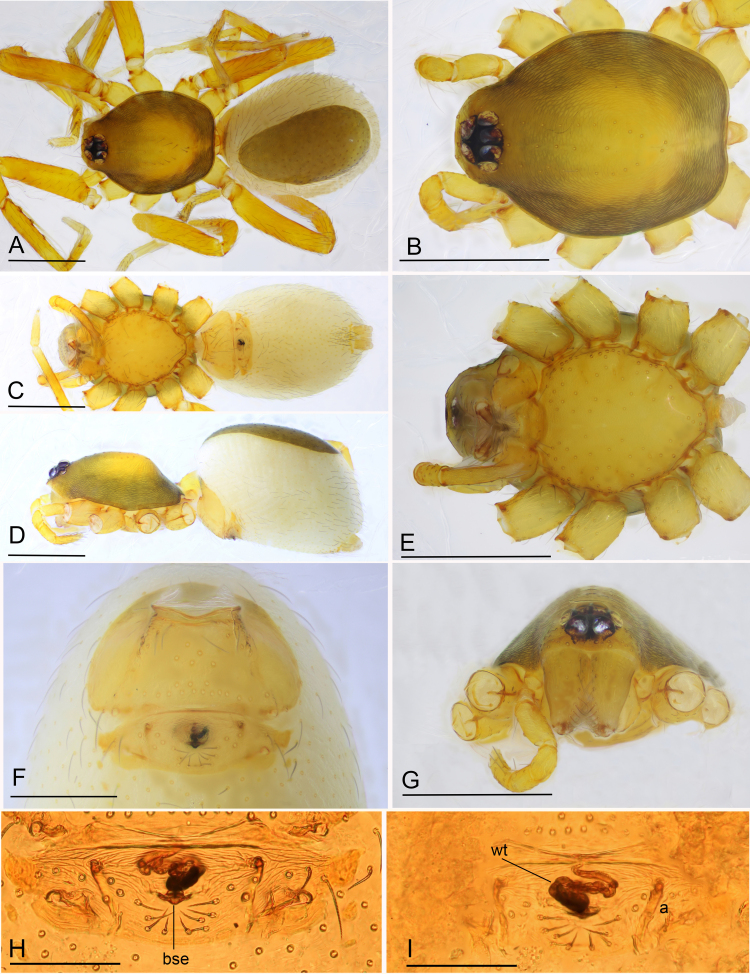
*Ischnothyreus yueluensis* Yin & Wang, 1984, female (SYNU-452) **A.** habitus, dorsal view; **B.** prosoma, dorsal view; **C.** habitus, ventral view; **D.** habitus, lateral view; **E.** prosoma, ventral view; **F.** epigastric region, ventral view; **G.** prosoma, anterior view; **H.** endogyne, ventral view; **I.** endogyne, dorsal view. Abbreviations: a = apodemes, bse = bowl-shaped sclerotised extension, wt = winding tube. Scale bars: 0.4 (A–E, G), 0.2 (F) and 0.1 (H, I).

**Female** (SYNU-452) (in mm): same as male, except as noted. Body: habitus as in Fig. 6A, C and D; body length 1.49. Carapace: 0.67 long, 0.53 wide. Mouthparts: chelicerae and endites unmodified. Abdomen: 0.84 long, 0.58 wide; dorsal scutum covering 1/2 of abdomen length, about 3/4 of abdomen width. Epigastric area: surface without external features. Endogyne (Fig. 6I): from middle of slightly thickened margin of postgastric scutum runs a dark, simple winding tube, ending in a small, narrow bowl-shaped sclerotised entension; posteriorly directed apodemes present.

#### Diagnosis

This species is similar to *Ischnothyreus concavus* Richard, 2016 in the stick-shaped sclerotized process on the male fang base, but can be distinguished by the prolateral lobe (Fig. 5I) of palpal bulb, which is lacking in *Ischnothyreus concavus* (Richard et al. 2016: fig. 33) and the epigastric area (unmodified (Fig. 6H) vs. with central goggle-shaped process (Richard et al. 2016: figs. 36B, C and D)).

#### Distribution

**Figure 7.**
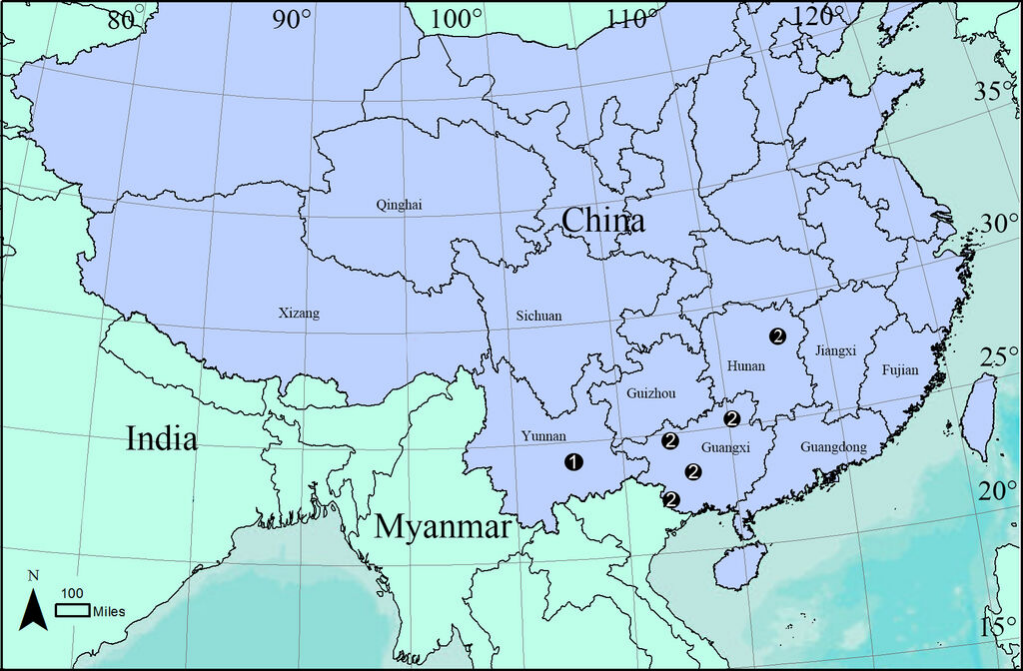
Distribution of two *Ischnothyreus* species from China. 1. *I. yunlong* sp. n. 2. *I. yueluensis* Yin & Wang, 1984.

China (Hunan, Guangxi) (Fig. 7).

## Supplementary Material

XML Treatment for Ischnothyreus yunlong

XML Treatment for Ischnothyreus yueluensis
